# Compound heterozygous variants in *MAN2B2* identified in a Chinese child with congenital disorders of glycosylation

**DOI:** 10.1038/s41431-022-01125-7

**Published:** 2022-05-31

**Authors:** Qi Tian, Li Shu, Chuqiang Shu, Hui Xi, Na Ma, Xiao Mao, Hua Wang

**Affiliations:** 1 Department of Obstetrics & Gynecology, Hunan Provincial Maternal and Child Health Care Hospital, Changsha, Hunan 410008 China; 2 National Health Commission Key Laboratory for Birth Defect Research and Prevention, Hunan Provincial Maternal and Child Health Care Hospital, Changsha, China; 3 Department of Medical Genetics, Maternal and Child Health Hospital of Hunan Province, Changsha, Hunan 410008 China

**Keywords:** Genetic counselling, Genetic testing

## Abstract

Congenital disorders of glycosylation (CDG) is a group inherited disorders. It is characterized by multi-organ dysfunction with significant morbidity and mortality. MAN2B2-CDG caused by pathogenic variants in the *MAN2B2* gene was a rare CDG. To date, only one case of MAN2B2-CDG was reported. The representative clinical features were immune deficiency, dysmorphic facial features, coagulopathy, and severe developmental delay. More cases are needed to support the pathogenesis of *MAN2B2* variation and elucidate its clinical heterogeneity. In this study, we described the clinical presentations of a CDG proband with compound heterozygous variants in *MAN2B2*. Serum N-glycan profiling was measured by MALDI coupled to time-of-flight mass spectrometry (MALDI-TOF MS). MALDI-TOF MS analysis of patient serum showed disorders of N-linked glycosylation, including increased N-glycans and elevated Man5/Man6 and Man5/Man9 value. Our proband presented severe developmental delay, dysmorphic facial features as in the previous case. But our case presented new features, including cleft palate and hypospadias with no immune deficiency. Our data expands both the molecular and clinical phenotypes of MAN2B2-CDG and highlights the importance of the role of *MAN2B2* gene in CDG.

Congenital disorders of glycosylation (CDG) are inherited metabolic diseases which was caused by variants in genes encoding enzymes involved in glycoprotein biosynthesis [1]. Clinical features of CDG are highly diverse which includes neurologic deficits, dysmorphism, immune disorders, hematologic abnormalities, and other malformations [2]. The majority of CDG are N-glycosylation disorders, and they are caused by enzyme deficiency or other malfunction in the N-glycosylation pathway [3].

MAN2B2 (MIM# 618899) is a core-specific α-1,6-mannosidase involved in lysosomal glycoprotein degradation. MAN2B2 cleaves the α-1,6-mannose residue and Man2GlcNAc1 to generate free monosaccharides. Normal MAN2B2 function is essential for source recycling in glycan synthesis [4]. To date, only one case of CDG-MAN2B2 was reported by Jan Verheijen et al. in 2020 [5]. The patient presented immune deficiency and severe developmental delay caused by a homozygous missense variant in the *MAN2B2* gene. The clinical features of the CDGs are usually highly heterogenic, however the clinical manifestations of N-glycosylation disorders caused by variants in the *MAN2B2* gene remain under-studied [3]. Here we presented a case with compound heterozygous variants in the *MAN2B2* gene, causing global developmental delay, cleft palate, and hypospadias but no immune deficiency. Abnormal mannosidase activity was confirmed by mass spectrometry analysis of N-linked and free glycans in the patient’s serum.

Our patient is the first child born to a non-consanguineous Chinese family. He is a 3 year and 5-month-old male born at full term with normal pregnancy. The birth weight was 2750 g, and the Apgar score was 9/10. He presented with feeding difficulties accompanied with nasal regurgitation and choking episodes because of the cleft palate. The cleft palate repairment surgery was performed at 6 months, and feeding difficulty got improved. He had poor head control and delayed ability to crawl. He was able to stand without support at 1 year old and has the ability to walk when he was 2 years old. He had a speech delay and could only make sounds like “mama”, “papa” at the age of three. Physical examination revealed facial dysmorphism, hypertelorism, small chin, upper cleft palate, bulging of occipital bone, hypospadias, hypotonia, and short stature (The photographs were concealed due to ethical reasons). Developmental delay was diagnosed, and the brain magnetic resonance image was normal. At the age of three, he started rehabilitation exercise.

Whole-exome sequencing (WES) and sanger sequencing validation were performed in the proband and his parents (Fig. 1). The proband was identified with MAN2B2 compound heterozygous variants NM_015274.1: c.440_442delCTC (p.Ser147del) (chr4:6588769-6588771), and c.2368G>A (p.Glu790Lys) (chr4:6612715-6612715). The father was a heterozygous carrier of the *MAN2B2* c.440_442delCTC variant, and his mother carried another variant c.2368G>A in the *MAN2B2*. The c.440_442delCTC variant is predicted by (The Human Splicing Finder system [6]) as an activation of a cryptic donor site and potential alteration of splicing. The variant c.2368G>A is evaluated by MutationTaster (v2.0), Combined Annotation Dependent Depletion (CADD) (v1.6), FATHMM (v2.3). The scores are 1, 34, −2.77, respectively, and the variant is predicted to be damaging or disease-causing. The allele frequency of c.440_442delCTC was 2.72e-4 in gnomAD database, 2.5e − 05 in the ExAC and 0 in the 1000 genomes database. The allele frequency of c.2368G>A was 5.4e-5 in gnomAD, 0 in the ExAC and the 1000 genomes databases.

**Fig. 1:**
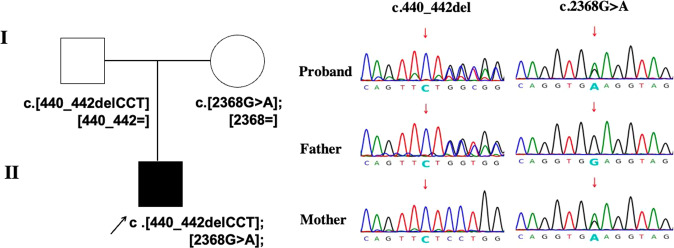
Pedigree chart and sanger sequencing results of the family. The pedigree with *MAN2B2* variants and sanger sequencing results of *MAN2B2* variants in family members.

The CDG laboratory tests were performed (Fig. 2 and Supplementary Table 1). The serum transferrin isoelectric focusing profiles were normal, and serum N-glycan profiles by MALDI-TOF MS showed a relative increase in the under-sialylated, mono-sialylated N-glycans and Man5/Man6 (0.95, normal range: 0.63–0.81). The previously reported case showed severe immune deficiency so we tested the immune profiles of our patient. He had normal serum IgG (8.38 g/L), IgA (1.26 g/L), IgM (2.23 g/L) and IgE (14.35 IU/mL) level. The cell count for B cells, CD3+ T cells (including CD3+ CD4+ and CD3+ CD8+ T cells) and NK cells were within the normal range. The blood routine tests were normal.

**Fig. 2:**
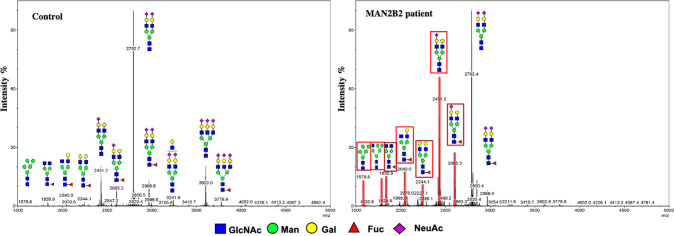
Hypoglycosylation of serum proteins in patient samples. N-linked glycans of serum from a representative healthy individual (left) and from patient with the *MAN2B2* variation (right) were analyzed by MALDI-TOF MS. Red boxes and peaks in the right panel indicate the increase of biantennary glycans.

MAN2B2 belongs to the mannosidase gene family, and it participates in the final steps of lysosomal glycoprotein degradation. The loss of enzymatic activities of other well studied family members, including lysosomal alpha-mannosidase (MAN2B1) and mannosidase beta (MANBA), resulted in alpha- and beta- mannosidosis [4, 7]. The clinical significance of MAN2B2 has not yet been fully validated. Only recently, were the expression profiles and enzymatic role of MAN2B2 revealed. MAN2B2 is an α1,6-mannosidase specific for cleavage of the α-1,6-mannose residue of N-linked glycans and cleaves the Chitobiase product Man2GlcNAc1 to generate Man1GlcNAc1[8]. Thus, the disorder of MAN2B2 results in the accumulation of glycans and causes the disorders of N-linked glycosylation. To date, only one case of MAN2B2 deficiency had been reported to cause a new type of CDG with severe growth delay, intellectual or developmental disability and immune deficiency, and MAN2B2-CDG case with genital and skeletal anomalies has never been reported [5].

Our study identified compound heterozygous variants in the *MAN2B2* gene in a pedigree. Although our proband shared some features with the reported MAN2B2-CDG, such as developmental delay or intellectual disability, he showed a few different clinical manifestations from the reported one. Our patients did not show a severe immune deficiency but with upper cleft palate, bulging of occipital bone and hypospadias. The different presentations between the two current cases are consistent with the heterogenic nature of CDG diseases [2, 8]. The useful diagnostic biomarker of MAN2B2-CDG was a loss of core fucosylation on serum N-glycans by mass spectrometry [9]. Both the previous case and our study showed abnormal mannosidase activity by MALDI-TOF MS analysis of N-linked and free glycans. And the primary clinical tool of transferrin isoelectric focusing profiles were negative in both cases. Therefore, genetic testing is considered reliable for determining the type of CDG, especially when the blood tests were normal [10]. The managements of MAN2B2-CDG were also different between these two cases. The reported case received hematopoietic stem cell transplantation to achieve stabilization of the disease, and our patient had cleft palate repairment surgery followed by rehabilitation exercise.

In summary, we reported here the second case of MAN2B2-CDG with developmental delay and multiple new features, including the regular immune system, upper cleft palate and hypospadias. Our study contributes to expanding both the molecular and clinical knowledge for this disorder and highlight the usefulness of genetic testing and MALDI-TOF MS analysis in N-glycosylation disorder.

## Methods

### Genetic investigation

Genomic DNA from peripheral blood leukocytes of the trio was extracted by Qiagen DNA Blood Midi/Mini Kit (Qiagen GmbH, Hilden, Germany). Whole exome sequencing (WES) was conducted on DNA from proband using a HiSeq 2500 system (Illumina) with a mean depth of 100×. Data are processed preliminarily according to the protocols of WES[12]. A public database (1000 Genomes Project, ExAC, gnomAD) was used to detect variants frequencies. The pathogenicity of variants was predicted using the following software programs: CADD [https://cadd.gs.washington.edu/]. MutationTaster [http://www.mutationtaster.org/]. FATHMM [http://fathmm.biocompute.org.uk/fathmmMKL.htm]. Sanger sequencing was performed on the DNA of the proband’s parents to validate the variants found in WES.

### N-glycans analysis of serum by MALDI-TOF MS

The samples were processed and analyzed as previously published [13]. In short, the N-glycans in the samples were released from serum glycoproteins using PNgase F, and released glycans were purified on porous graphitized carbon SPE columns, then permethylated and further purified on a preconditioned C18 Sep-Pak cartridge. Bruker UltraFlex II MALDI-TOF tested the purified permethylated N-glycans in reflective, positive mode with 500 *m*/*z* ~ 6000 m/z range. The data was analyzed by mMass software (Version 5.5.0). [10.1007/s10719-012-9376-3].

### Supplementary information

Supplementary Table 1

## Data Availability

The data that support the findings of this study are available from the corresponding author, Xiao Mao, upon reasonable request.
